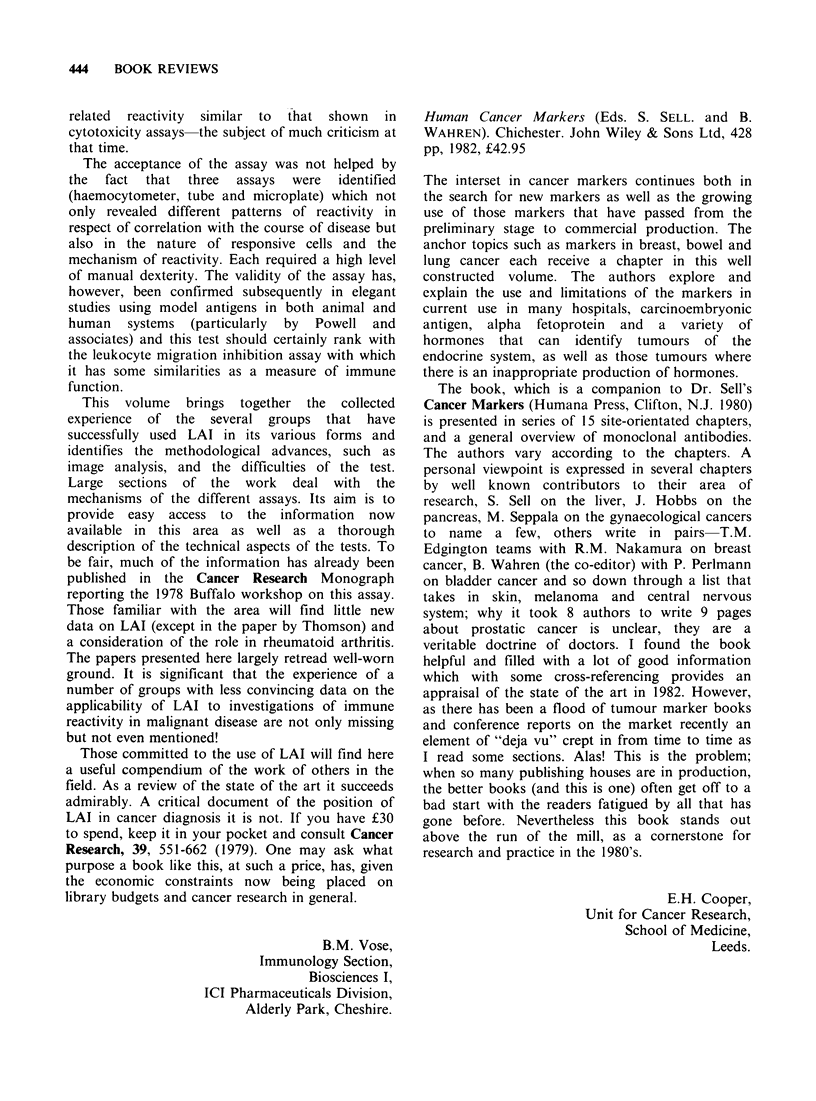# Human Cancer Markers

**Published:** 1983-03

**Authors:** E.H. Cooper


*Human Cancer Markers* (Eds. S. Sell. and B. Wahren). Chichester. John Wiley & Sons Ltd, 428 pp, 1982, £42.95

The interset in cancer markers continues both in the search for new markers as well as the growing use of those markers that have passed from the preliminary stage to commercial production. The anchor topics such as markers in breast, bowel and lung cancer each receive a chapter in this well constructed volume. The authors explore and explain the use and limitations of the markers in current use in many hospitals, carcinoembryonic antigen, alpha fetoprotein and a variety of hormones that can identify tumours of the endocrine system, as well as those tumours where there is an inappropriate production of hormones.

The book, which is a companion to Dr. Sell's **Cancer Markers** (Humana Press, Clifton, N.J. 1980) is presented in series of 15 site-orientated chapters, and a general overview of monoclonal antibodies. The authors vary according to the chapters. A personal viewpoint is expressed in several chapters by well known contributors to their area of research, S. Sell on the liver, J. Hobbs on the pancreas, M. Seppala on the gynaecological cancers to name a few, others write in pairs—T.M. Edgington teams with R.M. Nakamura on breast cancer, B. Wahren (the co-editor) with P. Perlmann on bladder cancer and so down through a list that takes in skin, melanoma and central nervous system; why it took 8 authors to write 9 pages about prostatic cancer is unclear, they are a veritable doctrine of doctors. I found the book helpful and filled with a lot of good information which with some cross-referencing provides an appraisal of the state of the art in 1982. However, as there has been a flood of tumour marker books and conference reports on the market recently an element of "deja vu" crept in from time to time as I read some sections. Alas! This is the problem; when so many publishing houses are in production, the better books (and this is one) often get off to a bad start with the readers fatigued by all that has gone before. Nevertheless this book stands out above the run of the mill, as a cornerstone for research and practice in the 1980's.

E.H. Cooper,
Unit for Cancer Research,
School of Medicine,
Leeds.